# Energy Expenditure and Metabolic Changes of Free-Flying Migrating Northern Bald Ibis

**DOI:** 10.1371/journal.pone.0134433

**Published:** 2015-09-16

**Authors:** Franz Bairlein, Johannes Fritz, Alexandra Scope, Ilse Schwendenwein, Gabriela Stanclova, Gertjan van Dijk, Harro A. J. Meijer, Simon Verhulst, John Dittami

**Affiliations:** 1 Institute of Avian Research “Vogelwarte Helgoland”, An der Vogelwarte 21, 26386, Wilhelmshaven, Germany; 2 Center for Organismic Systems Biology, Departments of Anthropology and Behavioral Biology, University of Vienna, Althanstr. 14, 1090, Vienna, Austria; 3 Clinic for Avian, Reptile, and Fish Medicine, University of Veterinary Medicine Vienna, Veterinärplatz 1, 1210, Wien, Austria; 4 Central Laboratory, University of Veterinary Medicine Vienna, Veterinärplatz 1, 1210, Wien, Austria; 5 Center for Behavior and Neurosciences (CBN), Rijksuniversiteit Groningen, Nijenborgh 7, 9747, AG, Groningen, The Netherlands; 6 Centrum voor Isotopen Onderzoek (CIO), Rijksuniversiteit Groningen, Nijenborgh 4, 9747, AG, Groningen, The Netherlands; 7 Waldrappteam, Schulgasse 28, 6162 Mutters, Austria, and Konrad-Lorenz Research Station, 4645, Grünau, Austria; Institute of Ecology, GERMANY

## Abstract

Many migrating birds undertake extraordinary long flights. How birds are able to perform such endurance flights of over 100-hour durations is still poorly understood. We examined energy expenditure and physiological changes in Northern Bald Ibis *Geronticus eremite* during natural flights using birds trained to follow an ultra-light aircraft. Because these birds were tame, with foster parents, we were able to bleed them immediately prior to and after each flight. Flight duration was experimentally designed ranging between one and almost four hours continuous flights. Energy expenditure during flight was estimated using doubly-labelled-water while physiological properties were assessed through blood chemistry including plasma metabolites, enzymes, electrolytes, blood gases, and reactive oxygen compounds. Instantaneous energy expenditure decreased with flight duration, and the birds appeared to balance aerobic and anaerobic metabolism, using fat, carbohydrate and protein as fuel. This made flight both economic and tolerable. The observed effects resemble classical exercise adaptations that can limit duration of exercise while reducing energetic output. There were also in-flight benefits that enable power output variation from cruising to manoeuvring. These adaptations share characteristics with physiological processes that have facilitated other athletic feats in nature and might enable the extraordinary long flights of migratory birds as well.

## Introduction

Nonstop flapping flights across large geographic barriers such as deserts or oceans during migration [1–3] are extreme and fascinating achievements, but the physiological adaptations associated with these feats are not thoroughly understood. Flight is energetically costly, and the key to the mystery may therefore be the birds’ abilities to meet the energetic costs and to cope with the negative impacts of long-term exercise. Costs and consequences of physical activity should increase with its effort and duration [4]. The availability of fuel along with a variety of other factors may change flight performance and could limit endurance. These other factors include the accumulation of lactate and oxidative damage, dehydration, electrolyte imbalance and acidosis, glycogen depletion, exceeding capacities for fat and protein catabolism, and oxygen debt. All are known to contribute to muscle fatigue and thus limit performance and duration. Following the typical mammalian model of exercise, one would expect flight to be accompanied by similar cost accumulations, fuel depletion, increased energy expenditure and exhaustion. This view is partly supported by studies on migrating birds but has become difficult to generalize in light of documented extremes in migratory performance, like the eight-day trans-Pacific flight of a Godwit [1], or the 3400 km trans-Atlantic flight of a Northern Wheatear [3].

In previous studies, the energetic costs of flight were described by employing aerodynamic models [5, 6], flying birds in wind tunnels [7–16], or by measuring energy expenditure in free-flying birds with calibrated heart-rate telemetry [17–19] and doubly labelled water (DLW) methods [20–23]. Physiological aspects of flight in birds was addressed by applying markers of exercise physiology developed in studies of humans [2] to birds at migratory stopover sites [2–39], to birds flying in a wind tunnel [40–42] or using homing pigeons [43–47]. While each of these studies produced valuable insights, they are paralleled by conflicting results and interpretations as well. Basic problems when comparing results were the lack of flight information, focus on a single aspect and time point of the response, and that species differing in size and flight modes were used. For example, in birds at migratory stopover sites nothing is known about the previous flight, and these birds may have been already at the site for an unknown period of time after landing before being trapped and examined. Only very few studies directly linked physiological markers to flight energetics, and considered flight-duration [12, 16].

Taking advantage of the framework of a human-led migration with access to the birds immediately prior and after a defined flight we aimed an integrated approach of using various markers for different metabolic processes along with the changes thereof during flight. We therefore combined simultaneously energetic measurements with an array of exercise physiology markers, enabling us to develop a more comprehensive picture of flight metabolism, with the ultimate aim to understand how birds achieve extreme long distance flights.

The hypothesis we investigate here is that physiological costs and cost accumulation can be compensated for during long flights. If this is so, then migratory birds may simply fly as far as their ecology dictates and their fuel reserves and body water requirements allow [16, 47–54].

In order to test our hypothesis, we introduced a new experimental approach to the study of the physiology of bird flight using human led flights to control the conditions under which the birds fly, and to take physiological measurements immediately before and after flights of defined lengths. We combined techniques to simultaneously document markers of energy expenditure, metabolite use (i.e. the change in concentration of plasma metabolites), respiration, water content and electrolyte balance. The techniques were then applied to defined flight performances in the Northern Bald Ibis (*Geronticus eremite;* NBI). NBI are migratory: documented migration routes are over 3000 km with individual non-stop flights of 500 km [55], and they show all the characteristics of the migrant models that have been used to date [56] including pre-migratory fattening as documented in this study (S5 Fig).

Our research was done within the framework of an autumnal human-led migration from SE Germany to central Italy with captive-bred birds [57]. The setup enabled us to document physiological reactions to flights of various lengths. Birds were hand-raised and trained to fly after an ultra-light aircraft, and land on command. It was possible to sample blood immediately before and after flights with exact knowledge of the intermediate performance. The birds’ flight speeds were regulated by the aircraft and held constant over time. In order to keep birds in continuous flapping-flight, migration bouts occurred in the morning before thermals had developed and would have the birds enforced to soar. Flight lengths were varied to relate energetics, fuel consumption and costs to exercise duration. We predicted that flight responses would reflect adaptations to oxygen debt, the change from glycogen to fat reserves as a muscle energy source and the accumulation of potentially adverse metabolic products. We initially analysed the results on the bases of all pre- to post-flight changes for the parameters. Thereafter we modelled the temporal patterns of pre-post changes with flight duration to document how energy expenditure, fuel use and cost accumulation had changed in-flight.

## Material and Methods

### 2.1. Methods

The project was carried out in the framework of a re-establishment program for the NBI in Central Europe (see www.waldrapp.eu). In the project, young NBI are hand-reared and trained to migrate to a wintering area in their first autumn. Birds are escorted along a migration route with microlight aircraft, from the German Prealps (Burghausen, 48°12.711' N, 13° 20.776' E) to central Italy (Laguna di Orbetello, 42°26.252' N, 11°11.645' E). The present study was conducted during the escorts of 2008. Flight training before migration enabled us to sample repeated flights of varying flight duration. Training began with first flight and continued until the flock was able to reliably follow the aircraft, taking off and landing on command. Training ended two weeks ahead of the start of the escorted migration flights in late August. To the end of the training period flight mode of the trained birds was similar to the one of a group of free-ranging birds at the Konrad Lorenz Forschungsstelle at Grünau, Austria. Escorted flights took place in the morning in order to avoid midday thermal turbulences. With this restriction, they were limited to about six hours. The end points of each flight were small recreation airports. After landing, an aviary was set up to house the birds until the next flight. Two double-seated microlights were used. A foster parent sat behind the pilot. Flight speed was maintained between 35 and 45 km/h. It was adjusted in-flight to the behaviour of the flock. Flight altitudes were 50 to 400 meters above ground at above levels of up to 1380 m depending on geological and meteorological conditions. Adverse winds constrained the microlights so flights only occurred on days with stable weather conditions. The total direct flight distance was 1162 km. The flights were planned in advance and dependent on the presence of airports along the route where the birds were allowed to land. Flight duration often deviated from plan as a result of weather conditions or the animals’ unwillingness to continue flight. In those cases, no blood samples were taken.

The analyses of in-flight physiology were based on comparisons of pre- and post-flight measurements. Prerequisites for data inclusion were; a) continuous flight between samples, b) successful documentation of flight duration and speed and, c) the exact coordination of the flight with the ground movement of apparatus and personnel necessary for immediate post-flight sampling. The specific characteristics of the flights and the numbers of individuals sampled are in the supplementary information (S1 Table). In 2008, there were 13 flights of which eleven were sampled successfully with durations of 65 to 205 minutes.

### 2.2. Sampling

Animals were sampled in the morning before take-off (on average (± SD) 43±13 minutes before flight), and immediately after landing. The lag between landing and individual sampling was on average 35±15 (3–69) minutes (S1 Fig). The times were noted and used to correct the data (see below). All birds were sampled during pre-migration as control values for the DLW analysis. Every other flight four to six other individuals were chosen per flight (S1 Table). Individual samples were separated by at least three days.

The birds were accustomed to handling from hatch. For blood collection, the bird was taken into the caretaker’s lap and its head was covered with a lightproof sack. For pre-flight measurements, blood was extracted from the *Vena jugularis dextra*. Post-flight samples were drawn from the cutaneous *Vena basilica*. The change in sample sources was to avoid hematomas and flight impairment after wing vein sampling. We are aware that the site of bleeding could have affected our measurements but there is no evidence in literature that there is a significant difference in haematological values between jugular and brachial vein values [58–59]. For determining resting energy metabolism a further blood sample was drawn the day after a flight. A 2.5 ml pre-heparinized syringe with a 25-gauge needle was used to collect 1.5 ml of whole blood. The handling-bleeding intervals were slightly longer post-flight but differences were not significant and not related to flight duration (S2 Table).

After collection, 200 μl capillaries with whole blood were flame-sealed for the doubly-labelled-water analyses and haematocrit tubes were filled for centrifugation. Thereafter, 65 μl of whole blood was injected into the CG4+ cartridge of a i-STAT blood analyzer, another aliquot was applied to an EC4 cartridge. The i-STAT is a commercial point-of-care device for blood chemistry parameters, validated in birds as well [60–62]. CG4+ was used to determine pH, pCO_2_ and pO_2_; EC4 for haematocrit (HCT), sodium (Na) and potassium (K). The remaining blood was centrifuged at 12.000 rpm for 10 minutes. Two plasma aliquots were prepared and frozen immediately for further analyses.

### 2.3. Physiological parameters

#### 2.3.1. Energy expenditure

Energy expenditure was measured using the DLW method [63–64] by a pre-flight injection of doubly labelled water (DLW) containing ^2^H and ^18^O isotopes (a standard mixture of 99.9% ^2^H water and 98.44% ^18^O water and a ratio of ^2^H to ^18^O of 3:2). Animals were held by the human foster parents and a 1ml DLW solution was injected in the pectoral muscle (*M*. *pectoralis profundus*). The injection times were noted (S3 Table). The injections occurred at least one hour before pre-flight blood sampling to ensure equilibration. The time point of injection had no effect on the data analysis because pre- and post-flight blood measurements were compared. The samples were stored at 5°C and later sent to the Centre for Isotope Research (University of Groningen). The analyses were done without knowledge of the flight protocol. The general procedure for analysis of ^18^O and ^2^H determination followed [65–67]. Briefly, after the blood was distilled in a vacuum line, δ^18^O and δ^2^H measurements were performed in automatic batches using a High Temperature pyrolysis unit (Hekatech) coupled to a GVI Isoprime Isotope Ratio Mass Spectrometer for the actual isotope analysis [68]. In the complete analysis scheme, several quality checks were incorporated, including the spread of initial values for similar situations, the spread of δ^2^H/δ^18^O ratios for initials and finals, and both absolute and relative differences. Local water standards (gravimetrically prepared from pure ^2^H- and ^18^O-water), covering the entire enrichment range of the blood samples, were applied for calibration purposes. Converting CO_2_ production into energy demands followed the calculations of Speakman [64] and Visser et al. [65] using a fixed respiratory quotient (RQ) of 0.8, in order to get comparable results to other published measurements of flight energy demands [20–23]. The error in the derived calorific equivalence of produced CO_2_ is less than 5% [64]. To assess energy expenditure for the flight only, corrections were made for the interval between sampling and take-off, and between landing and sampling. The corresponding post-flight resting expenditures were used as a proxy for the corrections. Total energy expenditure was estimated by determining the decay rate of isotopes between pre- and post-flight samples. Resting energy expenditure was calculated from the time interval between the post-flight sample and the recovery day sample.

#### 2.3.2. Blood chemistry parameters

Four groups of physiological parameters were chosen to study: 1) metabolites, 2) blood gases, lactate and phosphagens, 3) electrolytes and phosphates and 4) reactive oxygen metabolites (ROMs, a measure of oxidative damage). Metabolite analyses included plasma concentrations of triglycerides **TRIG**, free fatty acids **FFA**, glycerol **GLY**, ß-hydroxybutyrate **HBA**, glucose **GLUC**, total protein **TP**, uric acid **URIC** and **UREA**. With the exceptions of FFA and GLY, these were measured on a bench-top analyzer at the UVM [69–71], using a Roche Hitachi 911 Chemistry Analyzer (S4 Table). Two-level, daily quality controls were performed. FFA and GLY were measured manually in duplicate at the University of Vienna using the EnzyChrom Free Fatty Acid Assay Kit (BioAssay Systems) and the Sigma Aldrich Free Glycerol Determination Kit (FG0100).

As parameters for respiration we measured the whole blood partial pressures of oxygen and carbon dioxide (**pO2, pCO2**), plasma lactate **LACT**, lactatedehydrogenase **LDH** and creatine kinase **CK** activity. Oxygen and pCO2 measurements were done immediately after sampling with the i-STAT cartridge CG4+. Haematocrit was measured after centrifugation. Potassium **K** measurement is sensitive to storage and thus was done with the EC4 i-STAT cartridge after sampling [72]. The other plasma parameters were measured later with the Hitachi 911 in combination with an integrated ion-selective electrode (K and Na) and Roche Kit for inorganic Phosphates (S4 Table).

#### 2.3.3. Reactive oxygen metabolites

Reactive oxygen metabolites were measured at the University of Groningen following the protocol of Costantini et al. [73]. In short, oxidative damage was estimated as the plasma concentration of ROMs (primarily hydroperoxides, ROOH) and measured by means of the d-ROM Test (Diacron, Grosseto, Italy). Plasma (20 μl) was diluted in 400 μl of solution containing 0.01 M of acetic acid/sodium acetate buffer (pH 4.8) and *N*,*N*-diethyl-*p*-phenylenediamine as chromogen. After incubation at 37°C for 90 minutes, the pink coloured complex was measured with a spectrophotometer (Beckman Coulter Du530) at 505 nm and the concentration was determined by comparison with a reference. Results were expressed as mmol l^-1^ of H_2_O_2_. Serum antioxidant capacity (OXY) was measured by the OXY-Adsorbent test (Diacron, Grosseto, Italy). This kit uses a colorimetric determination to quantify the ability of the antioxidant barrier to cope with the oxidant action of hypochlorous acid (HOCl). Plasma (10 μl) was diluted 1:100 with distilled water. A 200 μl aliquot of a titred HOCl solution was incubated with 5 μl of the diluted serum for 10 min at 37°C. Then, 5 μl of the same chromogen solution used for the ROMs determination was added.

An alkyl-substituted aromatic amine solubilised in the chromogen is oxidised by the residual HOCl and transformed into a pink derivative measured at 490 nm.

### 2.4. Statistics

Changes between post- and pre-flight values in the blood characteristics were expressed as the difference between the post- und pre-flight values. In order to account for possible effects of the time lag between landing and bleeding on the measurements linear regression analyses were performed for each parameter. Though in only five of the 24 parameters the relationship between the parameter values and time lag were significant, we used the residual to correct for time lag effects in all post-flight plasma characteristics. In order to test the relationship between energy expenditure and flight duration and between changes in the plasma characteristics and flight duration, respectively, the post-/pre-flight difference values were used in a General Linear Repeated Measures Model approach with energy expenditure and plasma characteristics as dependent variables, flight duration as fixed factor, and the individual bird as a random effect and the sequence of flight (calendar date) as repeated measures. To visualize the relationship between post-flight changes in plasma characteristics and flight duration in all parameters for which GLM resulted in a significant relationship to flight duration, best-fit regression models were applied and the best-fit line is shown. All statistics were performed with SPSS 21.0 (IBM, New York). In all cases, *p* < 0.05 was considered significant.

### 2.5. Use of animals

The animals used in this work were obtained under licence from Zoo Vienna, Austria; Zoo Zurich, Switzerland; Zoo Prague, Czech Republic; Konrad-Lorenz Research Station, Austria; and Game Park Rosenegg, Austria. All experiments were under licence from and approved by the Bundesministerium für Wissenschaft und Forschung, Referat für Tierversuchswesen und Gentechnik, Vienna, Austria (BMWF-66.006/0014-II/3b/2010). Only animals in good health, as approved by a participating veterinarian, were used for flights. To reduce stress, the head of the bird was covered during manipulation and bleeding. The protocol was approved in the field by the Office of Advisory Committee for Animal Experiments, University of Veterinary Medicine, Vienna (protocol nr. 31/2009).

## Results and Discussion

### 3.1. Flight energy expenditure

**Fig 1:**
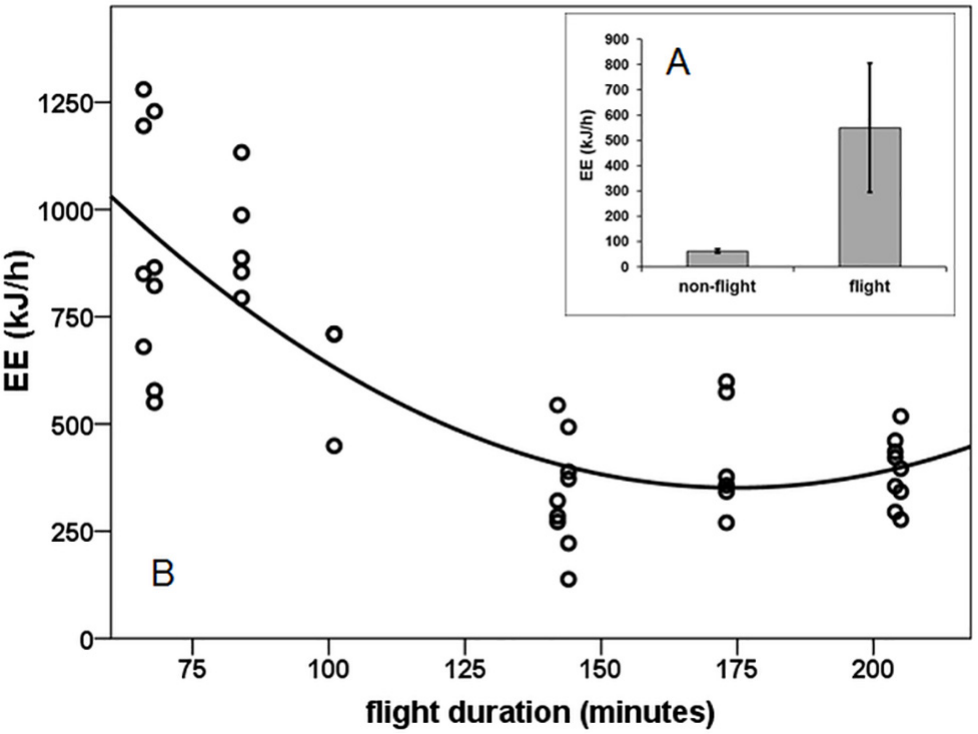
Energy expenditure during flight. **A**: Mean (± s.d.) energy expenditure (kJ h^-1^) during rest and during flight. **B**: Relationship between flight energy expenditure EE (kJ h^-1^) and flight duration.

Flight energy expenditure was significantly elevated above non-flight energy expenditure (Fig 1A; paired t-test: t_41_ = 12.307, p<0.001). Rate of energy expenditure decreased with flight duration but appears to level in longer flights (Fig 1B; GLMM, F = 12.969, p = 0.002). As flight durations were equally distributed over the season, and as energy expenditure did not correlate with calendar date (partial correlation between energy expenditure and date controlled by flight duration: r_par_ = -0.142, p = 0.383) training was not a factor in energy expenditure. As body mass did not significantly vary with flight duration (S2 Fig), mass specific energy expenditure (S3 Fig) decreased similarly with flight duration as did total energy expenditure (Fig 1B). Thus, the instantaneous costs of flying are lower in longer flights enabling the birds longer flight duration with the same amount of fuel. Total flight costs did not significantly increase with flight duration (S4 Fig; Person correlation: r = 0.220, n = 41, p = 0.168). One reason for increased energy expenditure in shorter flight could be, however, that the portion of taking off and climbing is relatively larger in shorter flights and progressively “dilute” as flight duration increases. Despite that, we did not observe any other modification of the birds’ flight behaviour over flight time. Consequently, the change could be related to corresponding changes in fuel metabolism and muscle efficiency.

### 3.2. Post-flight changes in plasma metabolites

**Fig 2:**
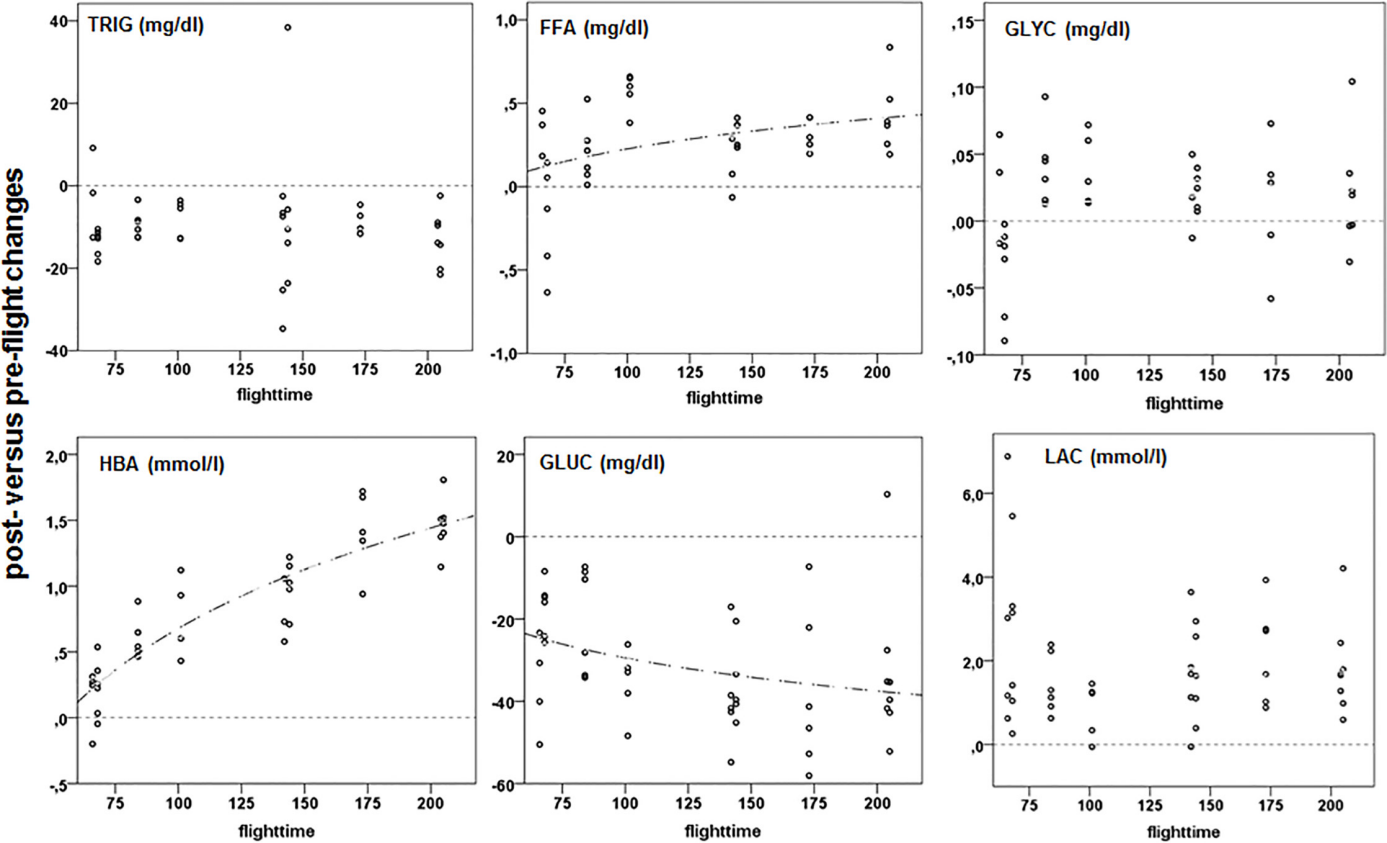
Post-flight versus pre-flight changes in plasma levels of fat and carbohydrate metabolites in relation to flight duration (flight-time in minutes). Best-fit regression lines are shown for significant relationships with flight duration. Horizontal dotted lines show the respective zero (no change) line.

As in many migrants, the NBI lost body fuel in the course of migration (S5 Fig) reflecting use of metabolic fuels. This use of fuels was reflected in the dynamics of metabolic markers. Post-flight plasma triglycerides (TRIG) were significantly lower than pre-flight, independent of flight duration (Fig 2; S5 Table). The use of TRIG from lipid reserves with increasing flight duration was also documented by the increased plasma levels of free fatty acids (FFA; Fig 2) and plasma glycerol (GLYC; Fig 2). The use of fat as energy substrate was finally illustrated by the increase in plasma levels of β-hydroxybutyrate (HBA; Fig 2), a by-product of lipid catabolism. HBA can have negative physiological effects via ketoacidosis which may explain the decrease of plasma pH with flight duration (Fig 4).

**Fig 3:**
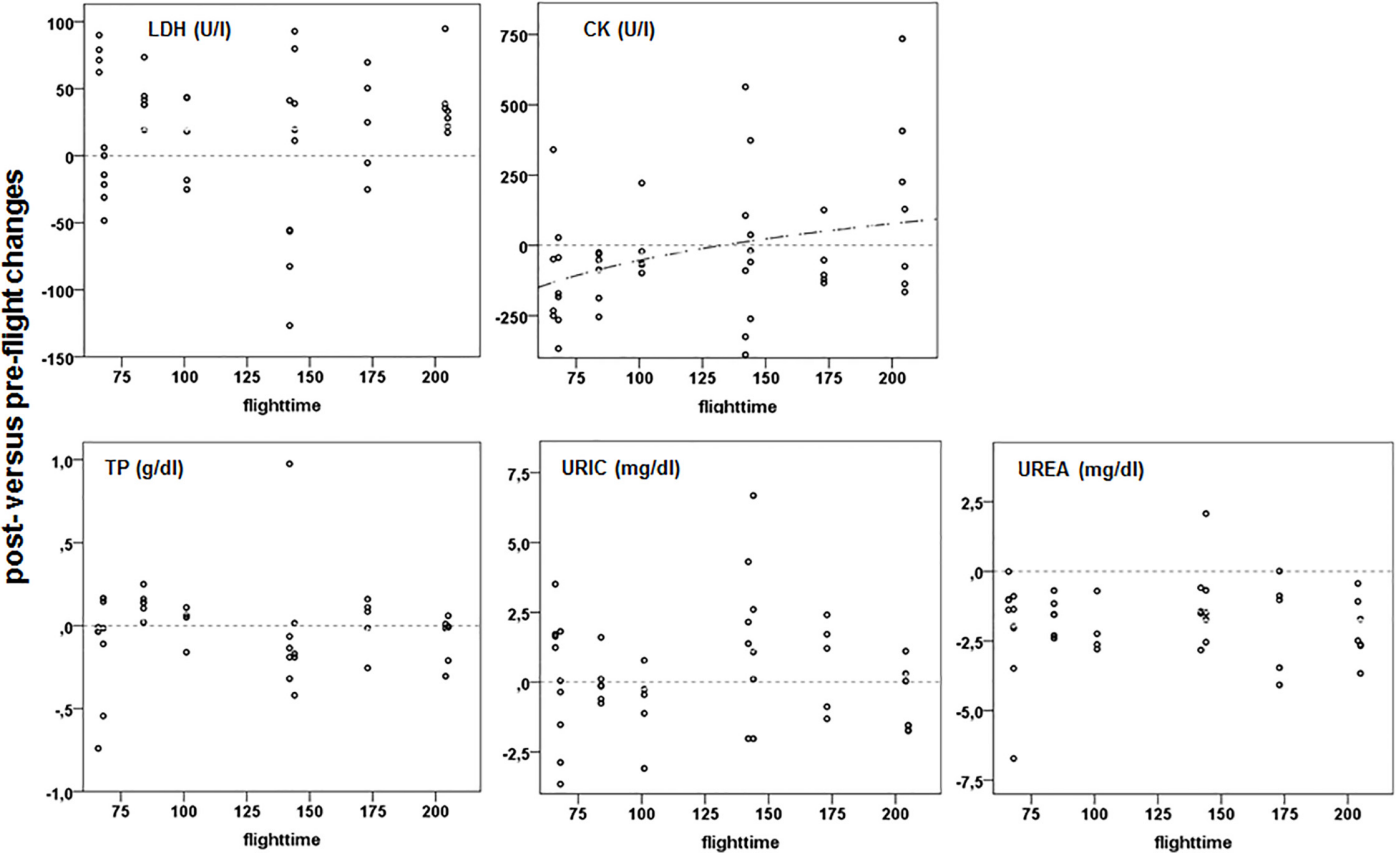
Post-flight versus pre-flight changes in plasma levels of LDH, CK and protein metabolites in relation to flight duration. For further explanation see Fig 2.

Post-flight plasma glucose (GLUC; Fig 2) levels were significantly lower than pre-flight levels and decreased with flight duration. Post-flight levels of markers for protein use and breakdown, plasma total protein (TP; Fig 3) and plasma uric acid (URIC; Fig 3), did not differ from pre-flight values, but TP slightly decreased with flight duration while URIC increased, reflecting the use of protein in addition to lipids similar to previous studies [25, 28, 39, 45]. However, post-flight plasma urea (UREA; Fig 3) levels were low compared to pre-flight levels, contrasting previous studies which reported elevated plasma UREA levels in homing pigeons immediately after return [45]. This difference may reflect differences in protein use during flight which might be less in migratory birds as compared to resident birds though trained for long homing flights.

### 3.3. Blood gases and products of anaerobic metabolism

**Fig 4:**
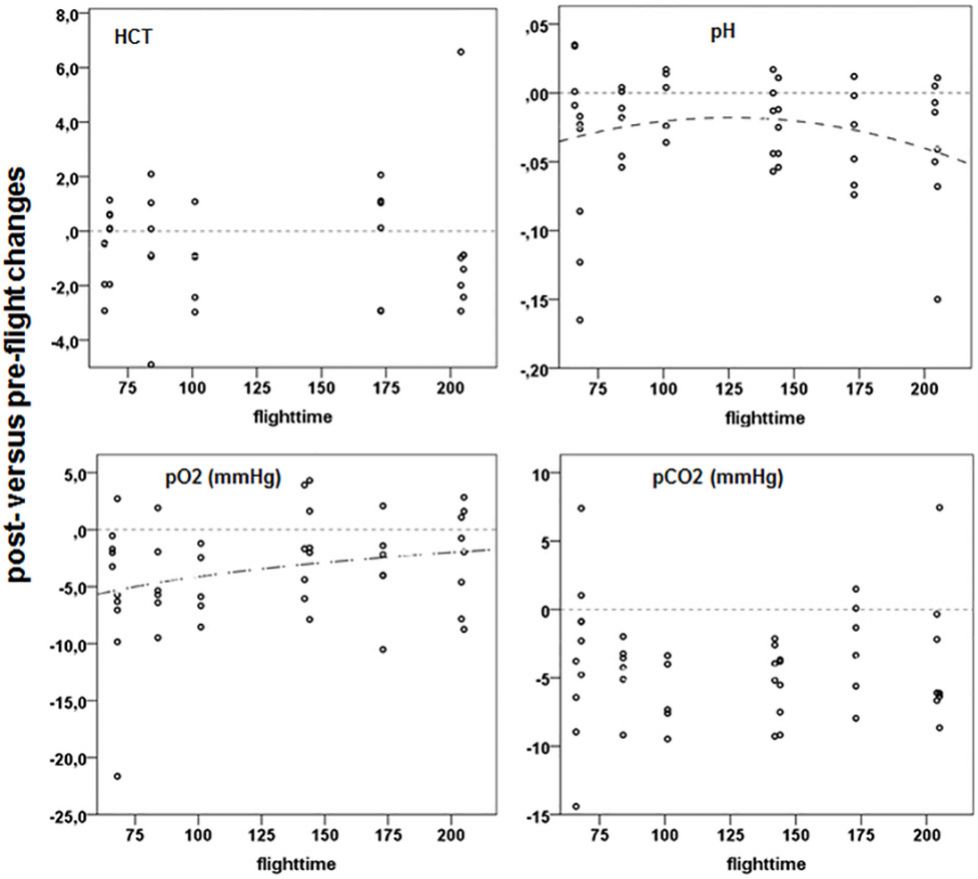
Post-flight versus pre-flight changes in plasma levels of HCT, pH and blood gases in relation to flight duration. For further explanation see Fig 2.

Blood gases, lactate and phosphagen kinase are parameters that interact with exercise, metabolite use and gas exchange. A prelude for consideration here is that bird flight can enhance ventilation with accompanying costs and benefits. Venous pO_2_ decreased after flight (Fig 4, S5 Table) in short flights but the decrease in pO_2_ decreased with flight duration indicating recovery from an oxygen debt with increasing flight time which might be due to increased ventilation. Venous pCO_2_ decreased after flight with no relationship to flight duration. The decrease in blood pCO_2_ may be a direct product of increased metabolism and respiration. Increased exhalation may decrease the potential for acidosis during exercise [74].

Post-flight lactate (LAC; Fig 2) differed significantly from pre-flight with elevated post-flight levels but irrespective of flight duration, while lactate dehydrogenase activity (LDH; Fig 3) significantly increased with flight-time (S5 Table). Lactate and lactate dehydrogenase activity are directly related to oxygen availability. Increased levels signal anaerobic glycolysis. For long, lactate was largely considered a dead-end waste product of glycolysis due to hypoxia, a major cause of muscle fatigue, and a key factor in acidosis-induced tissue damage but is at present seen as an important intermediary in numerous metabolic processes [75]. Consequently, increased plasma lactate levels do not necessarily reflect muscle fatigue and impaired exercise capabilities. Rather, lactate is considered to be substrate for oxidative pathways [76–78] which may facilitate long endurance flights. Plasma-LDH increases are from muscle and liver sources and thus related to both lactate production and metabolism.

**Fig 5:**
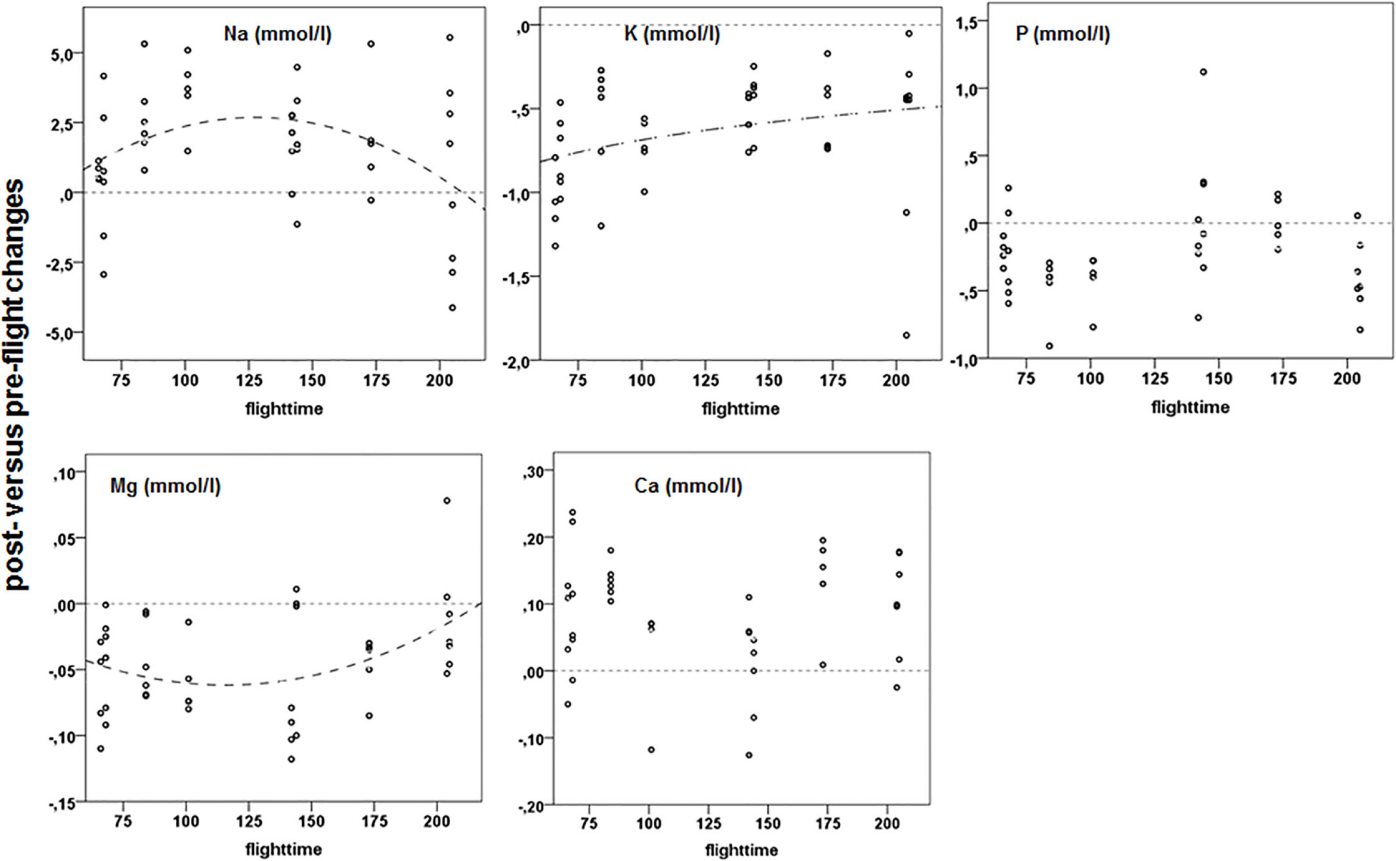
Post-flight versus pre-flight changes in plasma electrolytes in relation to flight duration. For further explanation see Fig 2.

Plasma creatine kinase levels (CK) did not change significantly during flight (Fig 3). This is in contrast to other studies which found CK increases in flight and was assumed them to reflect muscle damage and changes in membrane permeability [24, 79]. Our results do not support the muscle damage assumption although the few cases of birds with high post-flight CK levels (Fig 3) raise the possibility that muscle damage might exceptionally occur. Thus, migrants may avoid muscle damage behaviourally, or may have efficient biochemical and physiological defences against exercise induced muscle injury [24] Phosphate (P), a marker for CK activity and plasma P effects did not change during flight (Fig 5; S5 Table). All of the parameters are associated with processes that differ from aerobic catabolism and oxidative phosphorylation in terms of energy production. They are coupled to metabolic processes with very rapid and high production rates of ATP that are also costly. The negative side is that they reduce the total ATP yield [80]. There was evidence for the use of anaerobic pathways in-flight. As mentioned above, they also documented the presence of a high anaerobic threshold in Ibis similar to that found in well-trained human athletes [81].

We did not find a decrease in haematocrit (HCT; Fig 4; S5 Table) after flight as it has been reported in other studies [25, 42, 82]. However, data on changes in HCT in relation to flight and energy expenditure are controversial [83].

### 3.4. Post-flight changes in plasma electrolytes

Among electrolytes, sodium (Na), potassium (K), magnesium (Mg), and phosphates (P) were measured because of their relationships with exercise, blood gases and metabolism. Na increased and K decreased in short flights but recovered to normal levels with increasing flight duration (Fig 5; S5 Table). Both are paired in the sodium pump associated with a wide range of physiological functions from osmolarity to excretion. In effect, there were shifts among cellular, interstitial and plasma pools that occurred in-flight. The increase in Na is unlikely due to potential evaporative water loss because Mg decreased as well, besides K (Fig 5; S5 Table), and there was no change in HCT (Fig 4; S5 Table). Ca levels were significantly increased after flight, irrespective of flight time (Fig 5; S5 Table). In homing pigeons no changes in plasma electrolyte concentrations were found [45]. Although electrolytes serve a wide variety of functional purposes and their concentration and balance in intra- and extracellular fluids are tightly regulated is [84–85] we do not know much about changes due to exercise except their link to water balance. Therefore, we can only speculate about the conflicting results and the functional purposes. An increase in Na may support muscle function in terms of power output, cardiovascular performance, and lactate trafficking [84]. Similarly, the small but significant decreases in the plasma levels of K, Mg and P may have been connected to alterations in muscle activity as plasma volume did not increase. Phosphates interact directly with muscle power output and indirectly with it via interaction with haemoglobin affecting oxygen binding [79]. Similar decreases in the plasma free phosphate pool during exercise have been related to muscle uptake in mammal studies. For Mg, muscle uptake, to increase power output, and plasma decreases are common phenomena in exercise physiology [74]. With increasing flight duration these pre-/post-flight differences decreased indicating that the birds tapped into plasma stores of specific electrolytes to increase flight efficiency and lessen the impact of extended exercise.

### 3.5. Reactive oxygen metabolites

**Fig 6:**
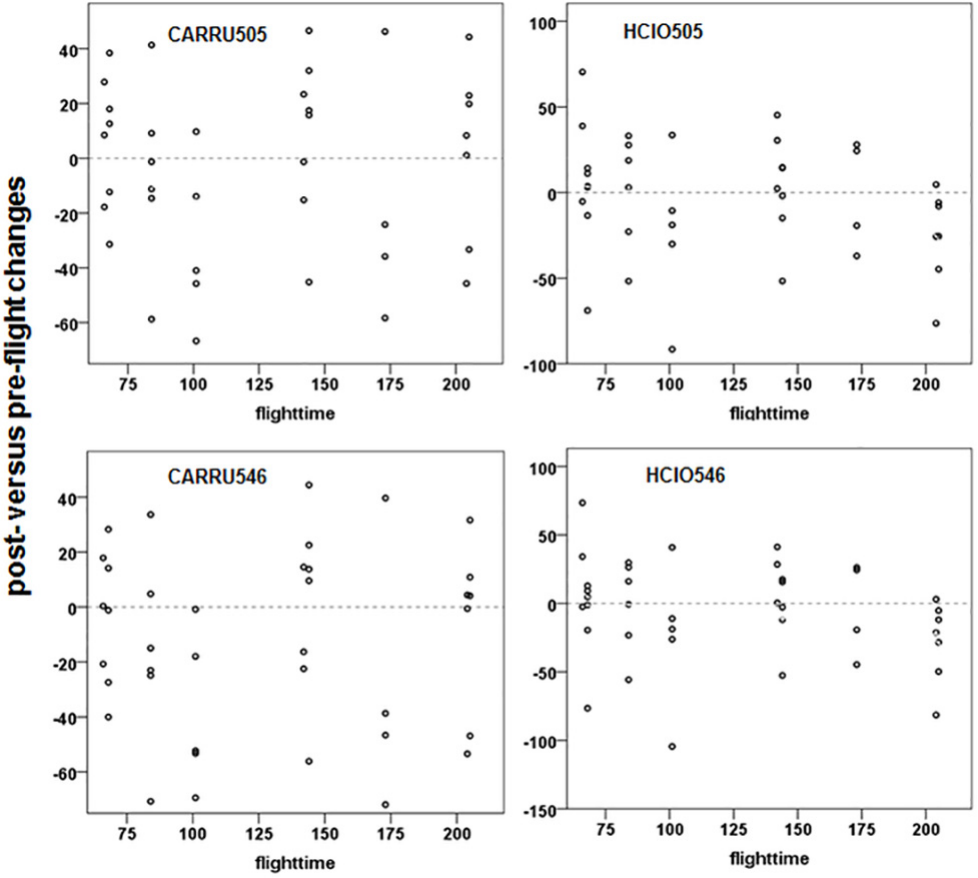
Post-flight versus pre-flight changes in plasma reactive oxygen metabolites in relation to flight duration. For further explanation see Fig 2.

Strenuous exercise is often seen as resulting in oxidative stress with increased oxygen consumption and free radical production [40, 86]. To examine potential oxidative stress we monitored serum reactive oxygen metabolites (ROMs) and total serum antioxidant capacity (OXY). Neither ROMs nor OXY changed significantly from pre- to post-flight nor in relation to flight duration (Fig 6; S5 Table). OXY was measured as the ability of the serum antioxidant barrier to cope with the oxidant action of hypochlorus acid. There were no correlations among ROMs, OXY and flight duration (Pearson correlation; p>0.5). This is in contrast to previous reports that flying birds are exposed to oxidative stress [40, 86]. Similar to Kestrels [87] NBI appear to be able to cope with oxidative stress but the effective defence system is unknown.

## Conclusions

By using an integrated approach with combining parallel measurements of energy expenditure and an array of exercise physiology markers in the Northern Bald Ibis we can draw conclusions which we believe are indicative of principles that make moderate and long-distance migratory flights possible. Our data support earlier results in flying pigeons [8] that during take-off and early flight the birds primarily use their carbohydrate (glycogen) stores to fuel their flying. This is the consequence of the need for more power output and the higher energy expenditure in early flight. As these glycogen reserves last not long [8] they appear to become somehow anaerobic rather shortly after take-off. They then went quickly into fat metabolism and continued so until the end of the flight. Utilizing fatty acids from lipolysis provided the birds with power and were part and parcel to metabolic burden. With the continued loss of GLUC (Fig 2) and the initial but stable depletion of plasma TRIG (Fig 2) and TP pools (Fig 3) fatty acids were mobilized from lipid stores and GLUC loss was perhaps compensated for, in part, with LAC and GLY metabolism [88]. Plasma sodium increases and body fluid changes occurred early in flight and they were an integral part of the physiological adaptation. They became more moderate with prolonged exercise.

The absence of a continuous LAC increase (Fig 2) documented the balance between accumulation and metabolism. LAC metabolism is part of a shuttling mechanism with both anabolic and catabolic products. Similar LAC patterns during exercise have been described in detail for mammals [74]. In line with recent ideas concerning its role in prolonged exercise [89] LAC shuttling might even be advantageous for the birds during flight. The hypothesis predicts that stores are used during exercise to compensate for GLUC decreases. Hence GLY, LAC and LDH interactions occurred that maintained or perhaps even improved exercise performance during flight. Thereafter, although the birds returned to aerobic metabolism with increasing flight duration they maintained an anaerobic capacity. Exercise around the anaerobic threshold is known to be an efficient exercise strategy in long distance human runners [74]. It is therefore not a surprising phenomenon in migrating birds.

Accumulations of HBA (Fig 2) could, in time, become a physiological burden for the birds. Within our flight durations we did find plasma pH changes, so ketosis may become a problem. However, the non-linear relationship between increase in post-flight HBA and flight duration hints on an asymptotic levelling in HBA levels in very long flights. However, the effects of ketone metabolism on brain function in birds are not known. Post-flight HBA increases are known in other migrants, and it is assumed that birds have a high systemic tolerance for them, similar to that for LAC [90]. CK is considered to be an indicator of strenuous exercise and muscle damage [24, 79]. In contrast to mammals, CK has been found to have positive or enhancing effect during exercise in other vertebrates like reptiles [91]. It may, therefore, also have had a specific physiological role in-flight. It was liberated or leaked from muscle and liver sources initially and remained elevated as flight continued. In muscle CK shunts ATP into a pool of CrP that increases the energetic base for contraction and lessens heat production. In this way, some of the negative impacts of FFA metabolism in muscle [92–93] can be avoided. In essence, the intramuscular CK effects have the potential of being a key element in the increased energetic efficiency of longer flights by balancing need and availability.

The study has provided insights to flight physiology that support our initial hypothesis that physiological stress and cost accumulations can be compensated for during flight and that as a consequence migratory species are not particular constrained by physiological processes. It would seem that they can fly as far as their ecology dictates and their fuel reserves and body water requirements allow. Though derived from studying a medium distance migrant these results may help to understand the extraordinary athletic feats of migrants crossing oceans and deserts [1–3] without intermittent stopping. They also have consequences for the understanding of the evolution of migration strategies [94] and open up questions about the physiology of endurance exercise that may have shaped the evolution of movement of many other animals as well.

## Supporting Information

S1 Data Master data set. (PDF)

S1 Fig Frequency distribution of time lag between landing and bleeding. (DOCX)

S2 Fig Pre-flight body mass (mean ± s.d.) of Northern Bald Ibis at the various flight durations. Pre-flight body mass did not vary with flight duration (ANOVA: n = 41, F = 0.417, p = 0.902).
(DOCX)

S3 Fig Mass specific energy expenditure (kJ h^-1^*g^-1^) during flight. (DOCX)

S4 Fig Total flight costs of Northern Bald Ibis in relation to flight duration. (DOCX)

S5 Fig Changes in fuel load of Northern Bald Ibis prior to and during “migration”. Fuel load is the proportion of body mass gained, calculated as (body mass–lean body mass)/lean body mass. Lean body mass is the minimum body mass prior to subsequent continuous body mass increase.
(DOCX)

S1 Table Statistics of flights with blood sampling. The number of birds sampled refers to the number of individuals that were injected with DLW and subsequently sampled for pre-flight parameters.
(DOCX)

S2 Table The time spans (in minutes) for bleeding of individuals (capture to blood) and DLW injections (capture to injection). Data shown reflect the samples where exact times were noted; hence the discrepancy in numbers of samples with the other tables.
(DOCX)

S3 Table Basic statistics of time points (ranges) for injections, pre-flight bleeds and recovery day DLW bleeds. Post-flight data varied with the length of the flight.
(DOCX)

S4 Table Summary of the kits and methods used to analyze blood chemistry parameters in NBI plasma with a Hitachi 911 Automatic Chemistry Analyzer. (DOCX)

S5 Table Summary statistics for the post- versus pre-flight comparisons (t-test; significant differences in bold) and the relationship between post-/pre-flight change and flight duration (Mixed Model; except for Ca and HCT with each 6 degrees of freedom, df was 8 in all other parameters; significant relationships in bold). (DOCX)
