# Supplementary material for: Energy Expenditure and Metabolic Changes of Free-Flying Migrating Northern Bald Ibis
Source: PLoS One. 2015 Sep 16;10(9):e0134433. doi: 10.1371/journal.pone.0134433 (PMC4573986; doi:10.1371/journal.pone.0134433)
Supplement: S1 Data — (PDF) [file pone.0134433.s001.pdf]

Master data set.

bird_ID	date	bodymass_1	distance	flighttime	bleed_interval	EE_flight	TRIG_0	TRIG_1	ffa_0	ffa_1	GLY_0	GLY_1	GLUC_0
1	17-Aug-2008	1.284	43	66	14	1.195	28	37	0,434	0,796	0,128	0,187	
1	21-Aug-2008	1.264	131	173	33	357	40	35	0,386		0,064	0,086	
1	27-Aug-2008	1.264	110	144			41		0,154		0,059		
1	7-Sep-2008	1.286	45	101									
2	21-Aug-2008	1.324	131	173	18	574		40		0,579		0,100	
2	27-Aug-2008	1.294	110	144	40	138	64	53	0,427	0,402	0,148	0,171	
2	7-Sep-2008	1.304	45	101	47	709	54	48	0,260	0,614	0,065	0,106	
2	12-Sep-2008	1.312	158	205				49		0,528		0,106	
3	21-Aug-2008	1.221	131	173	45	599	63	52	0,497	0,402	0,085	0,057	229
3	27-Aug-2008	1.220	110	144	30	389	69	45	0,253	0,424	0,057	0,052	220
3	7-Sep-2008	1.206	45	101	14	710	58	45	0,359	0,822	0,132	0,198	225
3	12-Sep-2008	1.200	158	205	56	396	65	50	0,454	0,614	0,156	0,156	213
4	17-Aug-2008	1.250	43	66	30	680	53		0,701		0,079		224
4	21-Aug-2008	1.202	131	173	55	342	60	49	0,496	0,553	0,056	0,107	226
4	27-Aug-2008	1.232	110	144	19	372	64	58	0,454	0,743	0,107	0,124	227
4	7-Sep-2008	1.208	45	101	34	449	54	50	0,281	0,662	0,107	0,107	242
4	12-Sep-2008	1.196	158	205	49	518	59	56	0,535	1,053	0,096	0,181	227
5	17-Aug-2008	1.232	43	66	23	850	55	53	0,645	0,865	0,068	0,095	215
5	21-Aug-2008	1.224	131	173	60	270	59	51	0,493	0,399	0,045	0,050	215
5	27-Aug-2008	1.224	110	144	34	222	16	54	0,464	0,493	0,050	0,068	
5	7-Sep-2008	1.196	45	101	40		55	50	0,389	0,778	0,036	0,050	236
5	12-Sep-2008	1.204	158	205	38	277	76	54		0,665	0,072	0,054	228
6	17-Aug-2008	1.394	43	66	39	1.280	54	41	0,938	0,868	0,167	0,135	228
6	21-Aug-2008	1.350	131	173	30	377	54	42	0,931	0,989	0,140	0,070	219
6	27-Aug-2008	1.368	110	144	12	493	64	50	0,535	0,827	0,075	0,081	231
6	7-Sep-2008	1.346	45	101	24		61	48	0,474	0,701	0,081	0,086	236
6	12-Sep-2008	1.346	158	205	62	342	63	42	0,812	0,602	0,011	0,005	228
7	19-Aug-2008	1.214	51	84	18		66	57	0,507	0,461	0,030	0,116	213
7	25-Aug-2008	1.196	115	142									
7	1-Sep-2008	1.224	142	204	12	435	64	50	0,708	0,886	0,101	0,066	226
7	10-Sep-2008	1.160	57	68	35	578	72	55	1,323	0,461	0,166	0,081	227
7	22-Sep-2008	1.190	125	193			83		0,788		0,126		233
8	19-Aug-2008	1.382	51	84	58	987	51	42	0,447	0,595	0,085	0,109	211

8	25-Aug-2008	1.384	115	142	59	321	72	46	0,453	0,356	0,109	0,073	210
8	1-Sep-2008	1.374	142	204	20	461	54		0,564		0,091		237
8	10-Sep-2008	1.356	57	68	59	1.229	54	42		0,322	0,115	0,073	205
8	22-Sep-2008	1.358	125	193			75	47	0,302	0,528	0,079	0,061	236
9	19-Aug-2008	1.156	51	84	50	1.133	50	46	0,585	0,374	0,056	0,068	210
9	25-Aug-2008	1.160	115	142	40	272	50	47	0,627	0,442	0,039	0,073	204
9	1-Sep-2008	1.162	142	204	5	295	52	43	0,468	0,803	0,141	0,135	212
9	10-Sep-2008	1.110	57	68	69	822	59	46	0,701	0,396	0,146	0,107	215
9	22-Sep-2008	1.132	125	193				59		0,805		0,225	
10	19-Aug-2008	1.402	51	84	32	794	54	43	0,714	0,782	0,064	0,096	221
10	25-Aug-2008	1.392	115	142	33	544	51	44	0,685	0,406	0,092	0,096	222
10	1-Sep-2008	1.408	142	204	38	355		58		0,768		0,119	
10	10-Sep-2008	1.330	57	68	51	865	70	51	1,259	0,511	0,206	0,096	224
10	22-Sep-2008	1.322	125	193									
11	19-Aug-2008	1.234	51	84	39	887	66	53	0,782	0,539	0,036	0,036	227
11	25-Aug-2008	1.236	115	142	28		93	58	0,588		0,058		204
11	1-Sep-2008	1.242	142	204			85	51	0,608	1,068	0,045	0,027	222
11	10-Sep-2008	1.192	57	68	22		60	47	0,621	0,532	0,047	0,036	222
11	22-Sep-2008	1.194	125	193			82		0,451		0,040		240
12	19-Aug-2008	1.292	51	84	46	854	66	53	0,443	0,360	0,101	0,095	221
12	25-Aug-2008	1.282	115	142	49	285	57	49		0,398		0,095	
12	1-Sep-2008	1.254	142	204	33	422	62	52	0,584	0,758	0,135	0,157	215
12	10-Sep-2008	1.202	57	68	41	550	55	44	0,870	0,471	0,146	0,101	223
12	22-Sep-2008	1.206	125	193			62		1,182		0,095		226

GLUC_1	FRUK_0	FRUK_1	HBA_0	HBA_1	UREA_0	UREA_1	URIC_0	URIC_1	TP_0	TP_1	LDH_0	LDH_1	CK_0	CK_1	Lac_0	Lac_1
	181	180	1,26	1,53	2,3	1,8	2,5	4,2	4,64	4,56	42	115	249	682	2,2	2,6
	166	172	0,43	2,05	1,8	3,0	2,4	5,9	4,78	4,36	47	142	127	471	2,1	5,5
	157		0,44		2,7		3,1		4,53		38		140		1,8	
		126		1,80		2,9		6,1		2,44		105		163		5,6
	143	129	0,45	1,55	4,3	3,2	4,7	7,1	3,16	2,79	53	95	282	285	1,8	2,8
	143	145	0,35	1,33	5,0	3,9	7,8	8,9	2,86	2,69	93	111	207	419	1,9	2,6
		127		1,90		4,3		6,2		2,69		108		707		3,6
192	127	136	0,42	1,63	0,3	0,9	7,3	10,5	3,00	2,76	59	144	274	438	3,4	3,7
210	145	138	0,34	0,96	1,4	1,8	7,2	8,3	3,45	2,88	40	102	246	385	2,4	4,5
197	121	112	0,36	0,75	1,0	0,8	6,7	6,9	2,83	2,60	62	116	148	176	2,0	3,0
193	128	126	0,48	1,83	0,0	0,3	5,4	5,5	3,10	2,81	57	128	430	929	1,5	2,4
184	120	110	1,35	1,06	1,9	1,6	2,7	7,2	2,83	1,94	49	162	166	114	2,4	8,8
204	133	130	0,41	1,92	4,4	2,3	3,8	4,3	3,10	2,91	62	99	121	432	2,4	3,2
194	129	126	0,28	1,20	3,0	2,2	6,9	5,5	2,95	2,87	43	77	154	317	2,2	3,0
222	135	133	0,40	0,90	4,1	2,7	5,5	7,4	2,88	2,76	87	88	169	325	3,6	3,0
192	125	123	0,50	1,83	4,3	2,4	3,8	3,7	2,92	2,67	41	96	188	437	2,5	2,7
183	127	124	1,27	1,47	3,6	3,4	5,3	7,7	2,88	2,73	45	134	336	439	1,6	2,4
214	137	134	0,52	1,28	5,6	4,3	6,6	7,7	2,97	2,83	76	97	390	681	1,6	3,4
208		143	0,10	1,15	0,7	4,0	0,6	8,4		3,22	13	132	133	731	0,2	2,6
212	140	133	0,48	1,29	5,4	4,6	6,5	6,7	3,01	2,92	66	115	188	674	1,7	1,4
206	114	112	0,32	1,61	5,7	4,4	5,6	5,3	3,05	2,65	38	89	245	359	1,5	5,1
211	118	118	1,16	1,29	3,8	5,2	2,8	5,8	2,64	2,41	58	167	164	189	1,6	4,0
183	129	138	0,61	1,93	2,4	2,6	3,5	5,7	2,67	2,63	91	139	144	221	2,1	2,5
190	131	149	0,43	1,42	5,4	4,1	3,1	6,1	2,87	2,62	47	136	164	224	1,5	1,7
	141		0,54		5,1		8,9	6,6	2,67		66	128	241	378	1,8	
207	113	116	0,58	2,20	4,4	4,0	4,8	5,3	2,70	2,45	59	140	113	357	1,6	1,2
212	123	124	0,43	0,84	1,6	0,7	4,9	7,1	2,90	2,95	44	96	72	160	1,5	3,6
195	127	131	0,41	1,52	0,0	0,0	4,7	5,4	3,05	3,00	36	140	124	429	1,8	3,3
225	142	137	0,58	0,70	0,1	0,0	6,9	7,7	3,11	3,10	46	79	206	465	1,8	1,5
	125		0,47		2,9		4,5		3,32		50		397		2,1	
221	122	135	0,54	1,25	4,6	6,0	3,9	5,2	2,67	2,63	38	127	87	216	1,9	1,6

189	138	131	0,34	1,22	4,4	3,7	5,9	10,0	2,83	2,47	136	125	348	349	1,6	2,5
	147		0,45		5,9		5,2		2,73		49		173		1,7	
200	129	118	0,41	0,59	4,7	0,1	3,4	5,4	2,38	2,23	97	94	186	209	1,8	1,9
200	127	127	0,31	1,37	3,7	0,9	6,0	8,8	2,70	2,43	44	148	148	544	2,2	1,9
219	129	136	0,83	1,22	4,5	3,9	3,4	4,9	3,50	3,39	53	165	607	912	1,6	2,1
201	178	148	0,74	1,35	2,4	2,4	2,9	5,6	3,74	3,35	155	227	459	1.287	1,7	4,7
172	150	141	0,57	1,93	3,0	2,1	3,9	4,1	3,85	3,52	51	90	214	654	2,2	3,4
215	148	138	0,64	0,97	4,3	3,3	4,1	3,5	3,82	3,64	46	68	287	572	1,3	3,5
184		133		1,65		2,2		4,6		3,59		106		1.085		2,5
198	127	123	0,60	0,98	4,1	3,7	4,2	4,5	2,73	2,59	39	83	101	125	1,4	1,8
191	144	133	0,60	1,08	3,2	3,8	3,6	9,0	3,05	2,75	122	92	226	354	2,0	2,6
209		144		1,63		4,1		6,6		2,92		168		882		2,1
226	132	142	0,77	0,65	4,0	3,8	4,3	7,8	3,67	2,87	58	83	214	286	1,3	1,9
207	133	138	0,76	1,15	4,7	3,8	4,8	6,2	3,04	2,95	46	114	253	424	1,3	1,8
159	132		0,40		3,9	3,4	7,8	6,7	2,87	2,41	158	97	243	103	2,4	1,9
191	140	131	0,40	1,67	5,0	3,2	7,4	6,7	3,10	2,76	71	136	243	461	1,2	4,1
215	135	134	0,69	0,88	3,2	3,1	5,9	5,1	3,08	2,86	57	74	243	345	1,5	4,3
	128		0,45		6,3		5,9		3,19		57		307		1,9	
209	130	134	0,43	0,94	4,6	5,1	7,0	8,4	2,87	2,80	28	105	132	384	2,3	3,8
195		139		0,79		4,7		13,5	2,01	2,74	258	169	411	841		2,4
199	131	143	0,34	1,75	6,2	4,9	7,6	9,8	2,93	2,75	109	173	274	1.227	1,9	3,8
229	138	121	0,69	0,52	3,2	2,7	5,3	3,0	2,80	2,58	73	83	224	312	1,7	6,5
	125		0,79		3,0		3,6		3,24		46		314		1,9	

Ca_0	Ca_1	P_0	P_1	Mg_0	Mg_1	Na_0	Na_1	K_0	K_1	HctZ_0	HctZ_1	pH_0	pH_1	pCO2_0	pCO2_1	pO2_0	pO2_1
2,64	2,63	1,7	1,5	0,72	0,69	141,0	142,0	4,3	3,2	44,0	43,5	7,508	7,513	36,2	30,0	48	47
2,66	2,57	1,6	1,7	0,71	0,71	141,0	144,0	3,5	2,9	42,0	39,0	7,494	7,479	34,7	27,3	45	46
2,50		1,3		0,68		139,0		3,8				7,501		32,5		43	
	2,17		1,7		0,78	138,0	144,0	3,5	3,4	44,0	45,0	7,400	7,416	28,1	29,9	46	44
2,32	2,20	1,5	2,0	0,69	0,73	139,0	145,0	3,3	3,1			7,426	7,477	33,5	25,0	45	48
2,32	2,25	1,4	1,3	0,77	0,76	140,0	146,0	3,7	3,3	45,0	44,0	7,467	7,531	32,7	25,9	47	43
	2,18		1,0		0,77		147,0		2,8		41,5		7,472		25,1		54
2,26	2,28	1,4	1,9	0,79	0,75	141,0		3,2	3,0	46,0	43,0	7,432	7,454	30,5	27,9	50	38
2,38	2,22	1,4	1,4	0,80	0,73	140,0	140,0	3,5	3,2			7,442	7,447	33,2	26,2	48	45
2,21	2,05	1,6	0,9	0,74	0,68	143,0	147,0	3,2	2,5	49,0	46,0	7,478	7,506	30,5	23,4	48	39
2,15	2,16	1,7	1,4	0,70	0,71	140,0	138,0	3,4	3,2	44,0	43,0	7,440	7,455	31,0	25,6	50	51
2,24	2,10	1,5	1,5	0,76	0,68	141,0	143,0	4,2	3,0	47,0	46,5	7,405	7,436	36,3	22,4	43	40
2,23	2,26	1,6	1,7	0,76	0,78	140,0	143,0	3,3	3,1	45,0	46,0	7,458	7,446	30,4	30,0	48	44
2,17	2,14	1,4	1,8	0,74	0,77	140,0	144,0	3,5	3,2			7,446	7,421	34,2	29,0	43	44
2,30	2,26	1,3	1,1	0,78	0,74	140,0	145,0	3,8	3,2	46,5	44,0	7,439	7,477	34,4	25,5	46	39
2,25	2,12	1,2	1,3	0,76	0,78	141,0	140,0	3,4	3,3	43,0	41,5	7,449	7,430	32,2	26,9	48	48
2,23	2,27	1,3	1,3	0,78	0,72	140,0	142,0	4,0	3,3	49,0	47,0	7,436	7,494	30,5	27,1	48	44
2,30	2,25	1,4	1,7	0,79	0,80	141,0	143,0	3,4	2,9	47,0	47,0	7,458	7,444	28,3	29,4	50	44
	2,30	0,2	1,5		0,77	140,0	143,0	3,7	3,1			7,474	7,454	31,3	28,2	52	43
2,36	2,31	1,2	1,2	0,77	0,73	142,0	145,0	3,8	3,4	48,0	49,0	7,453	7,469	29,8	27,1	50	42
2,14	2,17	1,5	0,9	0,70	0,73	140,0	141,0	3,4	3,5	46,0	43,5	7,463	7,351	27,7	35,8	52	42
2,23	2,24	1,5	1,4	0,77	0,78	140,0	142,0	3,8	2,9	48,0	45,0	7,443	7,516	32,7	24,4	47	44
2,10	2,19	1,6	1,9	0,78	0,78	140,0	143,0	3,7	3,1	42,0	44,0	7,409	7,451	32,3	27,2	46	41
2,21	2,22	1,5	1,3	0,75	0,76	139,0	141,0	3,6	3,4			7,439	7,439	30,3	26,7	47	45
2,22		1,4	1,1	0,76	0,77	139,0	145,0	3,9	3,0	45,0	44,0	7,454	7,442	30,7	27,1	45	43
2,04	2,03	2,0	1,8	0,72	0,75	139,0	139,0	3,2	3,0	43,0	42,0	7,434	7,507	31,0	23,4	48	44
2,20	2,25	1,7	0,9	0,73	0,74	138,0	144,0	3,4	3,2	43,0	44,0	7,460	7,460	31,7	26,9	42	35
						137,0		3,4				7,420		26,6		42	
2,24	2,30	1,2	0,9	0,72	0,81	138,0	144,0	3,3	2,9	49,0	48,0	7,443	7,441	34,6	28,7	48	43
2,28	2,29	1,2	0,9	0,83	0,84	138,0	142,0	3,9	3,0	47,0	47,0	7,467	7,479	30,4	30,1	49	38
2,30		1,3		0,77		142,0		3,5		49,0		7,447		32,0		51	
2,22	2,19	1,8	1,6	0,67	0,68	139,0	142,0	3,4	3,2	49,0	48,0	7,424	7,486	32,1	23,9	44	44

2,23	2,11	1,5	1,5	0,71	0,69	137,0	142,0	3,7	3,5			7,430	7,445	29,1	27,5	51	43
2,23		1,3		0,70		140,0		3,8		51,0		7,426		33,3		43	
1,99	1,86	1,2	0,9	0,61	0,65	139,0	142,0	3,3	2,6	43,0	43,5	7,478	7,511	26,9	25,6	49	40
2,09	2,07	1,5	1,3	0,69	0,71	141,0	140,0	3,7	3,0	48,0	47,0	7,454	7,518	34,9	25,1	44	51
2,33	2,36	2,1	2,0	0,78	0,76	141,0	145,0	4,1	3,1	51,0	46,0	7,416	7,420	35,0	32,3	41	34
2,35	2,34	1,8	1,8	0,79	0,74	139,0	142,0	3,6	3,0			7,450	7,433	35,1	30,6	47	44
2,46	2,42	1,7	1,3	0,75	0,76	141,0	144,0	3,8	2,7	48,0	46,0	7,446	7,401	34,1	32,0	53	45
2,31	2,34	1,5	1,6	0,77	0,76	140,0	143,0	3,6	3,2	46,0	47,0	7,447	7,393	34,2	36,4	50	42
	2,25		1,7		0,71		142,0		2,9		47,0		7,446		30,3		45
2,16	2,20	1,5	1,3	0,69	0,66	140,0	143,0	3,6	3,4	44,0	43,0	7,448	7,481	30,3	27,6	44	41
2,24	2,20	1,5	1,7	0,76	0,69	138,0	142,0	3,5	3,2			7,435	7,455	33,1	29,7	49	
	2,27		1,4		0,74	138,0	143,0	4,4	2,7	40,0	46,5	7,458	7,489	27,3	27,6	44	42
2,18	2,25	1,5	1,2	0,72	0,73	141,0	140,0	3,8	3,1	45,0	45,0	7,447	7,481	35,4	31,5	49	41
							140,0		3,0		47,0		7,469		30,8		47
2,43	2,44	1,4	1,3	0,78	0,75	140,0	144,0	4,0	3,4	49,0	49,0	7,417	7,445	33,2	29,6	44	37
2,15	1,94	1,6	1,0	0,77	0,68	140,0	141,0	3,1	2,8			7,420	7,465	33,7	24,9	51	54
2,31	2,36	1,2	1,0	0,78	0,75	141,0	145,0	3,5	3,0	49,0	48,0	7,423	7,452	31,8	27,6	51	40
2,33	2,25	1,2	1,6	0,78	0,71	139,0	144,0	3,7	3,2	47,0	45,0	7,459	7,395	32,3	31,8	49	51
2,31		1,4		0,70		142,0	143,0	3,7	2,9	50,0	47,0	7,412	7,467	34,7	28,4	50	51
2,34	2,32	1,5	1,4	0,70	0,74	139,0	144,0	3,7	3,5	47,0	49,0	7,443	7,435	34,6	33,4	46	35
	2,29		1,3		0,71	137,0	141,0	3,6	3,2			7,468	7,517	28,2	26,9	49	43
2,29	2,29	1,2	1,4	0,75	0,73	141,0	144,0	3,2	2,9	50,0	47,0	7,460	7,498	32,3	26,2	47	47
2,28	2,21	1,3	1,6	0,72	0,76	140,0	140,0	3,9	3,6	45,0	45,5	7,479	7,355	30,2	38,3	52	29
2,40		1,4		0,70		140,0		3,6		49,0		7,469		32,3		52	

CARRU505_0	CARRU505_1	CARRU546	CARRU546_1	umolHClOml505_(	umolHClOml505_1	umolHClOml546_0	umolHClOml546_1
127,52	110,17	123,28	106,35	187,20	256,85	186,50	259,98
174,09	130,13	172,37	127,12	181,88	128,02	178,15	125,60
147,02	158,20	142,37	154,32	175,18	142,54	167,62	141,50
169,59	135,14	165,08	130,69	133,00	158,55	131,61	157,93
155,65	174,06	151,59	169,31	131,72	144,41	128,12	143,87
149,08	82,82	145,24	79,63	145,44	53,11	142,92	38,50
133,57	155,13	130,69	150,00	165,75	154,73	165,01	153,18
187,75	131,13	186,61	129,66	222,96	182,95	226,26	181,67
128,15	175,33	123,81	173,39	175,34	172,42	173,72	170,97
193,21	148,51	190,42	146,44	178,91	166,44	175,40	164,36
172,85	141,06	171,10	137,54	182,85	154,62	182,82	154,48
180,79	189,98	178,98	185,59	151,62	189,23	154,19	188,40
148,01	125,66	146,19	123,81	179,07	156,48	171,28	152,11
131,37	164,40	129,41	161,19	193,94	177,19	188,11	176,32
182,28	142,55	180,51	139,07	138,69	170,07	124,29	165,28
131,87	177,32	130,42	172,37	203,80	156,95	204,77	155,11
159,19	188,25	156,36	184,83	142,73	135,36	136,55	134,18
143,79	190,98	142,12	189,92	154,69	177,40	152,46	176,95
170,36	186,51	168,81	185,85	152,29	166,34	146,72	164,20
178,56	165,40	175,68	164,24	186,27	166,05	184,35	165,62
162,42	187,25	156,86	184,58	172,93	163,64	166,04	160,94
134,85	144,54	132,71	142,37	172,98	205,13	161,41	191,17
152,24	160,93	151,27	158,90	179,27	183,26	170,42	173,47
170,61	140,31	168,81	138,31	180,01	189,19	178,60	187,85
147,24	147,77	143,39	144,18	146,05	161,66	144,41	160,65

165,64	166,17	162,70	162,43	176,91	218,93	176,83	218,19
	145,14		141,27	217,36	145,25	219,10	142,65
115,95	151,45	112,96	147,88	152,54	154,14	150,40	151,16
162,67	152,98	160,68	151,27	186,99	161,40	184,10	160,94
150,75	175,33	147,46	172,88	169,46	169,60	168,13	168,57
127,15	128,39	125,34	126,10	193,53	167,57	187,76	166,45
169,87	159,69	166,78	158,14	184,29	184,21	183,48	182,58
185,89	228,22	182,01	224,34	175,58	122,10	174,28	118,61
183,79	169,59	179,37	165,87	131,11	159,78	128,22	156,77
	164,07		159,79		157,46		156,41
141,72	155,92	138,36	151,06	169,54	153,37	169,08	149,71
150,39	136,99	146,83	132,54	194,95	195,77	195,58	194,95
	156,18		151,59		160,69		159,47
145,92	138,56	141,27	134,13	198,51	148,36	198,22	145,29
138,56	157,23	133,86	153,97	156,44	169,44	155,01	167,88
	162,75		157,41		167,19		165,85
192,99	135,67	189,68	131,48	136,53	161,74	134,06	160,53
199,56	154,86	195,50	151,06	197,64	119,44	197,63	116,21
137,51	177,21	134,13	173,54	161,65	162,56	156,99	161,78
