# Supplementary figures and images for: Energy Expenditure and Metabolic Changes of Free-Flying Migrating Northern Bald Ibis
Source: PLoS One. 2015 Sep 16;10(9):e0134433. doi: 10.1371/journal.pone.0134433 (PMC4573986; doi:10.1371/journal.pone.0134433)

**S1 Fig.: Frequency distribution of time lag between landing and bleeding**.

**
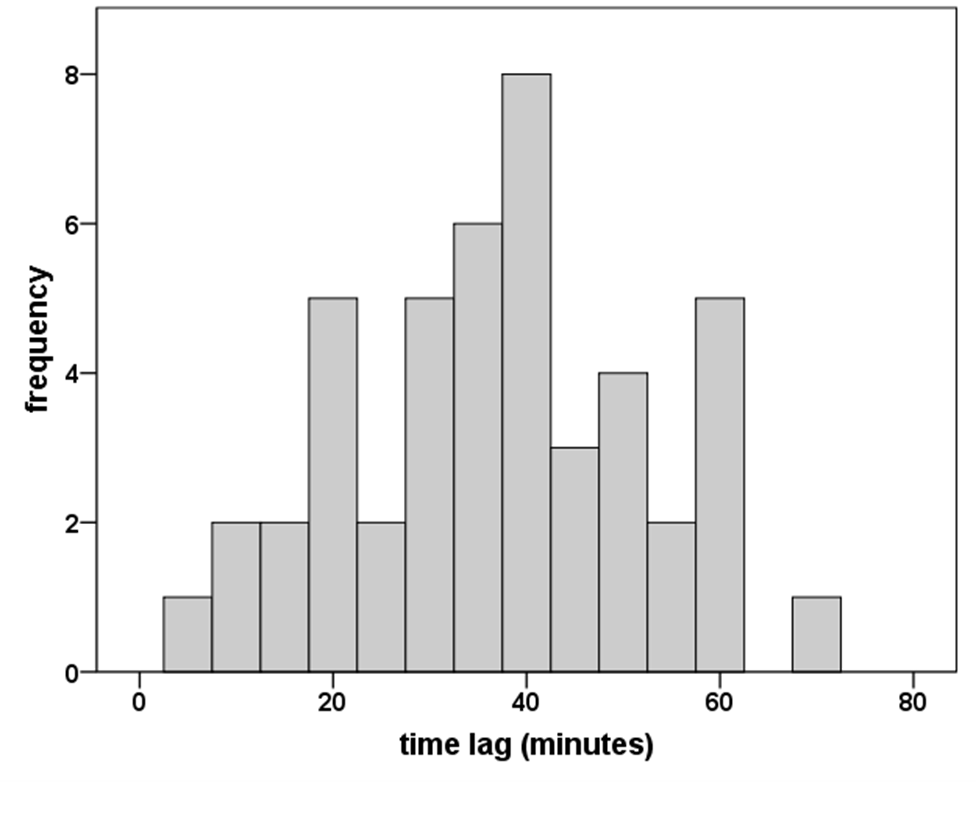
**

Supplement: S1 Fig — (DOCX) [file pone.0134433.s002.docx]

**S3 Fig.: Mass specific energy expenditure (kJ h-1*g-1) during flight.**


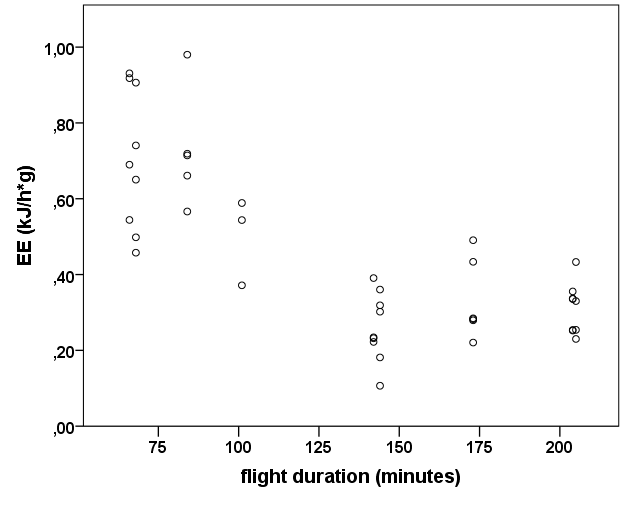

Supplement: S3 Fig — (DOCX) [file pone.0134433.s004.docx]

**S4 Fig.: Total flight costs of Northern Bald Ibis in relation to flight duration.**


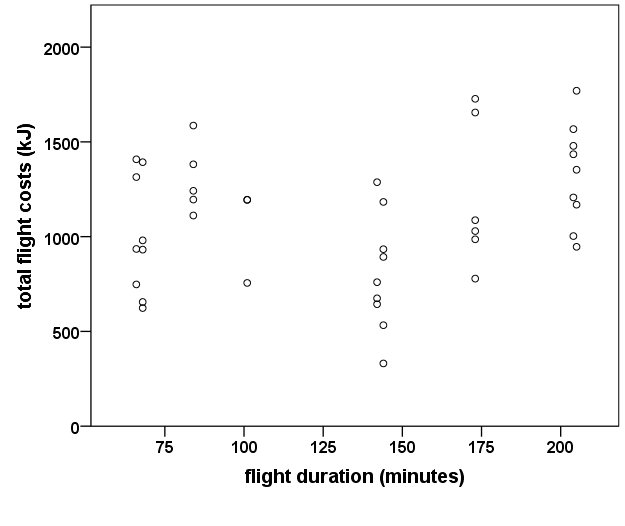


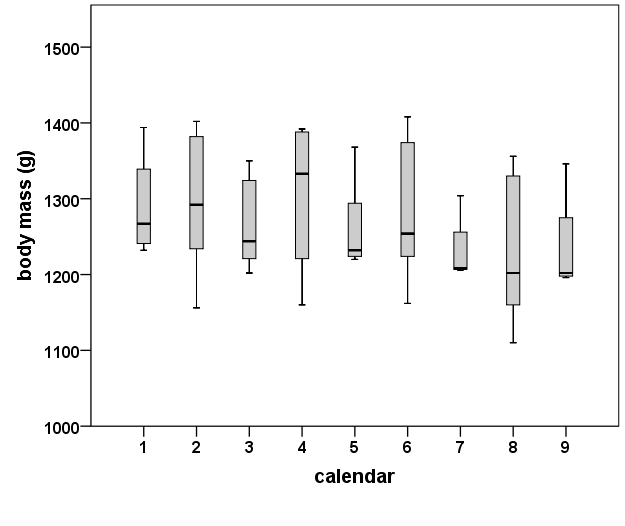

Supplement: S4 Fig — (DOCX) [file pone.0134433.s005.docx]
