# Supplementary material for: Energy Expenditure and Metabolic Changes of Free-Flying Migrating Northern Bald Ibis
Source: PLoS One. 2015 Sep 16;10(9):e0134433. doi: 10.1371/journal.pone.0134433 (PMC4573986; doi:10.1371/journal.pone.0134433)

**S2 Fig.: Pre-flight body mass (mean ± s.d.) of Northern Bald Ibis at the various flight durations.** Pre-flight body mass did not vary with flight duration (ANOVA: n=41, F=0.417, p=0.902).


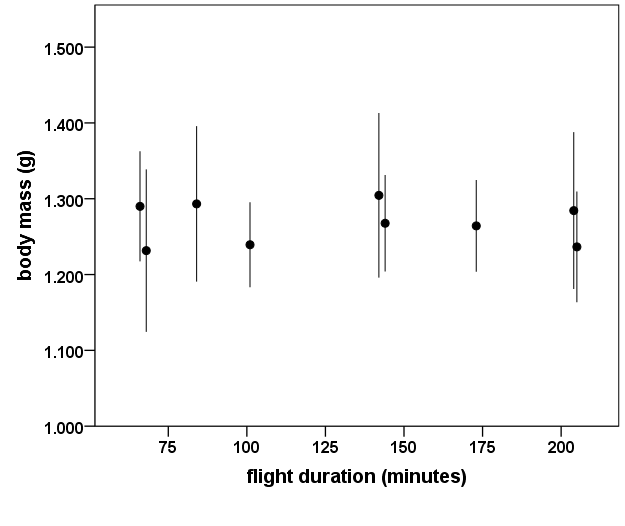

Supplement: S2 Fig — Pre-flight body mass did not vary with flight duration (ANOVA: n = 41, F = 0.417, p = 0.902). (DOCX) [file pone.0134433.s003.docx]
