# Supplementary material for: Energy Expenditure and Metabolic Changes of Free-Flying Migrating Northern Bald Ibis
Source: PLoS One. 2015 Sep 16;10(9):e0134433. doi: 10.1371/journal.pone.0134433 (PMC4573986; doi:10.1371/journal.pone.0134433)

**S5 Fig.: Changes in fuel load of Northern Bald Ibis prior to and during “migration”.** Fuel load is the proportion of body mass gained, calculated as (body mass – lean body mass)/lean body mass. Lean body mass is the minimum body mass prior to subsequent continuous body mass increase.


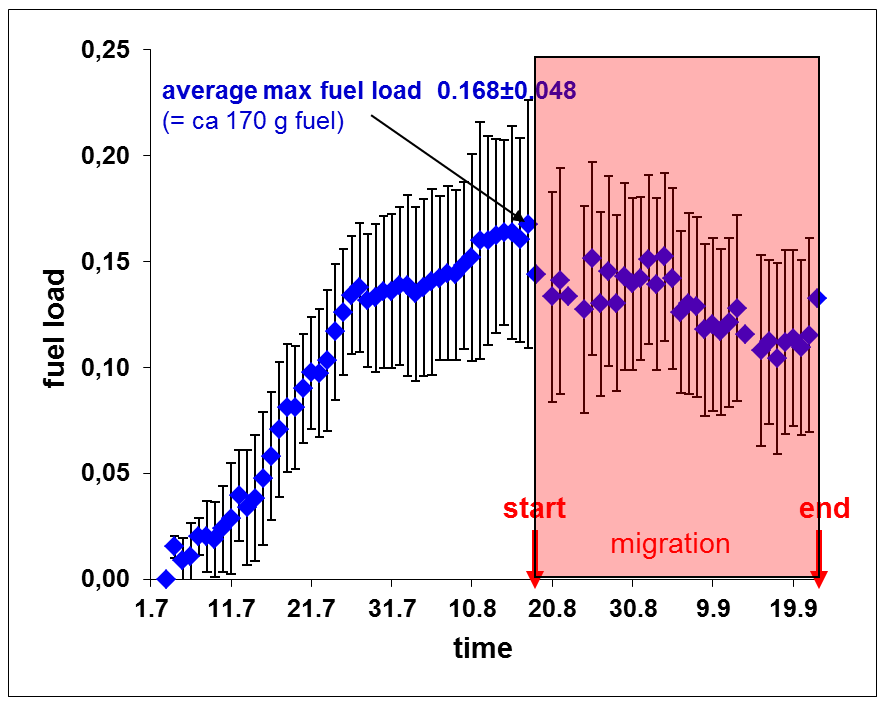

Supplement: S5 Fig — Fuel load is the proportion of body mass gained, calculated as (body mass–lean body mass)/lean body mass. Lean body mass is the minimum body mass prior to subsequent continuous body mass increase. (DOCX) [file pone.0134433.s006.docx]
