# Supplementary material for: Energy Expenditure and Metabolic Changes of Free-Flying Migrating Northern Bald Ibis
Source: PLoS One. 2015 Sep 16;10(9):e0134433. doi: 10.1371/journal.pone.0134433 (PMC4573986; doi:10.1371/journal.pone.0134433)
Supplement: S1 Table — The number of birds sampled refers to the number of individuals that were injected with DLW and subsequently sampled for pre-flight parameters. (DOCX) [file pone.0134433.s007.docx]

**Table S1:** Statistics of flights with blood sampling. The number of birds sampled refers to the number of individuals that were injected with DLW and subsequently sampled for pre-flight parameters.

| **Date** | **Flight distance** | **Flight time (min)** | **Speed (km/h)** | **Birds sampled** |
| --- | --- | --- | --- | --- |
| August 17 | 43 | 66 | 39.09 | 4 |
| August 19 | 51 | 84 | 36.43 | 6 |
| August 21 | 131 | 173 | 45.43 | 6 |
| August 25 | 115 | 142 | 48.59 | 6 |
| August 27 | 110 | 144 | 45.83 | 6 |
| September 1 | 142 | 204 | 41.76 | 6 |
| September 7 | 45 | 101 | 26.73 | 6 |
| September 10 | 57 | 68 | 50.29 | 6 |
| September 12 | 158 | 205 | 46.24 | 5 |
|  |  |  |  |  |
|  |  |  |  |  |
