# Supplementary material for: Energy Expenditure and Metabolic Changes of Free-Flying Migrating Northern Bald Ibis
Source: PLoS One. 2015 Sep 16;10(9):e0134433. doi: 10.1371/journal.pone.0134433 (PMC4573986; doi:10.1371/journal.pone.0134433)
Supplement: S2 Table — Data shown reflect the samples where exact times were noted; hence the discrepancy in numbers of samples with the other tables. (DOCX) [file pone.0134433.s008.docx]

**Table S2:** The time spans (in minutes) for bleeding of individuals (capture to blood) and DLW injections (capture to injection). Data shown reflect the samples where exact times were noted; hence the discrepancy in numbers of samples with the other tables.

|  | **n** | **Mean** | **SD** |  |  |  |  |  |  |
| --- | --- | --- | --- | --- | --- | --- | --- | --- | --- |
| Pre-flight | 47 | 5.94 | 1.96 |  |  |  |  |  |  |
| Post-flight | 42 | 8.10 | 2.47 |  |  |  |  |  |  |
| DLW Injection | 49 | 2.42 | 0.55 |  |  |  |  |  |  |
