# Supplementary material for: Energy Expenditure and Metabolic Changes of Free-Flying Migrating Northern Bald Ibis
Source: PLoS One. 2015 Sep 16;10(9):e0134433. doi: 10.1371/journal.pone.0134433 (PMC4573986; doi:10.1371/journal.pone.0134433)
Supplement: S3 Table — Post-flight data varied with the length of the flight. (DOCX) [file pone.0134433.s009.docx]

**Table S3:** Basic statistics of time points (ranges) for injections, pre-flight bleeds and recovery day DLW bleeds. Post-flight data varied with the length of the flight.

|  | **n** | **Begin** | **End** |
| --- | --- | --- | --- |
| Injection Times | 54 | 5:45 – 6:25 | 6:08 – 6:35 |
| Pre-flight Bleed | 51 | 7:04 – 7:27 | 7:30– 7:54 |
| Recovery Day DLW Samples | 27 | 8:12 – 8:38 | 8:42 – 9:17 |
