# Supplementary material for: Energy Expenditure and Metabolic Changes of Free-Flying Migrating Northern Bald Ibis
Source: PLoS One. 2015 Sep 16;10(9):e0134433. doi: 10.1371/journal.pone.0134433 (PMC4573986; doi:10.1371/journal.pone.0134433)
Supplement: S4 Table — (DOCX) [file pone.0134433.s010.docx]

**Table S4:** Summary of the kits and methods used to analyze blood chemistry parameters in NBI plasma with a Hitachi 911 Automatic Chemistry Analyzer.

| **Parameter** | **Kit #** | **Method** |
| --- | --- | --- |
| ß-Hydroxybutyrate. **HBA** | Randox RANBUT  D-3 Hydroxibutyrate | Kinetic enzymatic  3-hydroxibutyrate dehydrogenase |
| Calcium. **Ca** | Calcium. Roche # 1 148 92 16 | Chromogenic test with α-Kresolpthalein |
| Cholesterol. **CHOL** | Cholesterin CHOD-PAP  Roche # 1 148 92 32 | enzymatic color test  Cholesterinesterase/Trinder reaction |
| Creatinkinase. **CK** | Creatinkinase liquid IFCC  Roche # 1 213 25 24 | IFCC; substrate: Creatinphosphate; activation with N-acetylcysteine |
| Fructosamin. **FRUC** | Fructosamin. Roche # 193 00 10 | Colorimetric test Nitrotetrazolium blue. Ketoamines > Formazan |
| Glucose **GLUC** | Gluco-quant. Roche # 144 75 13 | Enzymatic UV-Test. Hexokinase |
| Lactate. **LACT** | Lactate. Roche # 1 182 28 37 | Enzymatic color test. Lactateoxidase |
| Lactatdehydrogenase. **LDH** | Lactatdehydrogenase liquid IFCC  Roche # 0300 20 98 | UV-Test; 37°C  Substrate: Lactate |
| Magnesium. **Mg** | Magnesium. Roche # 148 93 30 | Colorimetric endpoint; Xylidilblue |
| Phosphate. **P** | Anorganic Phosphate  Roche # 148 93 48 | Colorimetric endpoint; specimen blank;  Ammoniummolybdate |
| Tryglycerides. **TRIG** | Triglyceride GPO-PAP  Roche # 148 88 72 | Enzymatic color test Lipoproteinlipase/ 4 Aminophenazone |
| Total Protein. **TP** | Total-Protein. Roche # 155 38 36 | Colorimetric Test. Biuret –reaction |
| Uric acid. **URIC** | Uric acid plus. Roche # 166 18 50 | Enzymatic color test  Uricase / 4-Aminophenazone |
| Urea. **UREA** | Urea/kinetiv UV-Test  Roche # 1 148 93 64 | Enzymatic - kinetic UV-Test  Urease |
