# Supplementary material for: Energy Expenditure and Metabolic Changes of Free-Flying Migrating Northern Bald Ibis
Source: PLoS One. 2015 Sep 16;10(9):e0134433. doi: 10.1371/journal.pone.0134433 (PMC4573986; doi:10.1371/journal.pone.0134433)
Supplement: S5 Table — (DOCX) [file pone.0134433.s011.docx]

**Table S5:** Summary statistics for the post- versus pre-flight comparisons (t-test; significant differences in bold) and the relationship between post-/pre-flight change and flight duration (Mixed Model; except for Ca and HCT with each 6 degrees of freedom, df was 8 in all other paremeters; significant relationships in bold).
